# Loss of presenilin 2 function increases susceptibility to kainate‐induced status epilepticus and attenuates associated increases in hippocampal kainate‐type glutamate receptor expression

**DOI:** 10.1002/alz.091627

**Published:** 2025-01-03

**Authors:** Larissa Robinson‐Cooper, Stephanie Davidson, Rami Koutoubi, Melissa Barker‐Haliski

**Affiliations:** ^1^ University of Washington, Seattle, WA USA

## Abstract

**Background:**

Presenilin 2 (PSEN2) is one of three deterministic risk genes that increases the risk of early‐onset Alzheimer’s Disease. People with PSEN2 variants have increased risk of unprovoked seizures versus age‐matched unaffected individuals yet few studies have interrogated the contributions of PSEN2 on seizure susceptibility. Critically, PSEN proteolytic capacity may be a novel regulator of hippocampal kainate‐type glutamate receptors (KARs), with PSEN deletion reducing KAR availability and synaptic transmission in vitro (Barthet et al 2022). Kainic acid (KA) is a naturally occurring agonist for KARs that evokes sustained, severe seizures and status epilepticus (SE). We thus hypothesized PSEN2‐KO mice would demonstrate reduced KA‐SE latency, increased SE severity, worsened outcomes 7‐days later, and have altered hippocampal KAR expression versus similarly treated WT mice.

**Method:**

Using a repeated low‐dose systemic KA administration model to evoke SE, we quantified the latency to SE and extent of SE‐induced neuropathology in 3–4‐month‐old male and female PSEN2‐KO versus WT mice (n = 10‐16 mice/group/sex). GluK5, a KAR subunit, expression was colocalized in astrocytes and neurons by immunohistochemistry 7 days after KA‐SE or sham to define the impacts of PSEN2 deletion and SE on hippocampal KAR expression.

**Result:**

Regardless of sex, PSEN2‐KO mice were more susceptible to KA‐SE than WT mice. Male PSEN2‐KO mice progressed to SE faster than WTs (78.6±25.3 versus 98.2±16.0 min; t = 2.43, p = 0.027); female PSEN2‐KO mice were also more susceptible to KA‐SE relative to WTs (85.3±32.2 versus 112.2±15.4 min; t = 2.45, p = 0.022). Basal GluK5 expression did not differ between sexes or genotypes. However, in male mice 7‐days post‐KA‐SE insult GluK5 expression was significantly increased over basal levels in WT mice (p = 0.0028), but PSEN2‐KO mice GluK5 levels were unchanged from basal expression (p = 0.9819). Regardless of genotype, female mice GluK5 levels were unchanged from basal expression at 7‐days post‐KA‐SE insult, revealing sex differences in post‐SE outcomes.

**Conclusion:**

Loss of normal PSEN2 function increases susceptibility to KA‐induced seizures and SE, which cannot be explained by basal GluK5 expression differences. However, increased GluK5 expression in male WT relative to PSEN2‐KO mice 7‐days post KA‐SE, further substantiates a role of PSEN2 in KAR availability. Sex differences in GluK5 and GFAP were also apparent post‐SE.

